# Causes and consequences of divorce in a long‐lived socially monogamous bird

**DOI:** 10.1111/ele.14471

**Published:** 2024-12-31

**Authors:** Frigg J. D. Speelman, Terry Burke, Jan Komdeur, David S. Richardson, Hannah L. Dugdale

**Affiliations:** ^1^ Groningen Institute of Evolutionary Life Sciences University of Groningen Groningen The Netherlands; ^2^ School of Biological Sciences Macquarie University Sydney New South Wales Australia; ^3^ School of Biosciences University of Sheffield Sheffield UK; ^4^ Biological Sciences University of East Anglia Norwich UK; ^5^ Nature Seychelles Mahé Republic of Seychelles

**Keywords:** *Acrocephalus sechellensis*, age, mate fidelity, mate switching, partnerships, reproductive success, sex specific, Seychelles warbler, social monogamy, widowing

## Abstract

In socially monogamous species, sexual selection not only depends on initial mate choice but also mate switching. To date, studies lack assessment of (1) differences between passive (widowhood) and active (divorce) mate switching, (2) longer term fitness consequences (beyond the season post‐divorce) and (3) how age masks reproductive costs and benefits of divorce. We investigated causes and short‐ and long‐term consequences of mate switching and their age dependence using longitudinal data on Seychelles warblers (*Acrocephalus sechellensis*). Young and old males, but not females, divorced most frequently. Divorce propensity declined with pair‐bond duration and reproductive success in both sexes, but mate switching did not incur short‐term costs. Divorcees did not gain short‐ or long‐term fitness benefits compared to non‐divorcees. Rather, female early‐life divorcees that lost their breeding position had lower survival than females that never divorced. Divorce is likely a strategy to escape poor‐quality partnerships, but not all divorcees benefit from divorcing.

## Graphical abstract

In socially monogamous species, sexual selection involves both initial mate choice and mate switching. We found that divorce in Seychelles warblers occurs most among young and old males, and in pairs with short pair‐bond duration and low reproductive success. Ultimately, divorce does not confer short‐term fitness costs nor long‐term fitness benefits and even negatively impacts female survival.

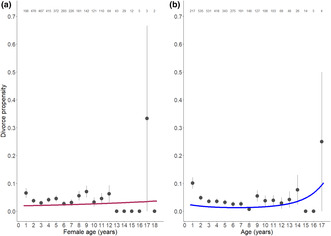

## INTRODUCTION

Partnership duration in socially monogamous species varies massively, ranging from lifetime fidelity (Black, 1996, 2001; Reichard & Boesch, 2003) to a single breeding attempt (Ludwig & Becker, 2008; Poirier et al., 2003; Veiga, 1996). Staying with the same partner can improve breeding success through increased familiarity and coordination of parental duties, and reduced mate‐searching effort (Black, 2001; Culina et al., 2020; Dreiss & Roulin, 2014). Alternatively, mate switching creates opportunities for attaining higher quality partnerships and breeding conditions, which can benefit individual reproductive success and survival (Black, 1996). Additionally, it can augment reproduction by enabling a second brood with a new partner within the breeding season (Béziers & Roulin, 2016; Roulin, 2002). Pair‐bond duration and mate‐switching rate depend on the frequency of widowing (one partner dies) and divorcing (both ex‐partners are alive but not paired with each other).

Divorce occurs in over 90% of socially monogamous species (Jeschke & Kokko, 2008) and has been associated with increased breeding success (Culina, Radersma, et al., 2015) and survival (Culina et al., 2013; Jankowiak et al., 2018; Nicolai et al., 2012). Understanding the causes (e.g. poor breeding success) and consequences (e.g. increases in breeding success and survival) of divorce can shed light on whether divorce is adaptive, the underlying processes driving mating strategies, and how mating systems are shaped by evolution (Reichard & Boesch, 2003). If divorce is adaptive, divorce should be triggered by poor partnership conditions or reproductive outcomes (Dubois & Cézilly, 2002), and/or the prospect of better conditions elsewhere (Black, 1996; Choudhury, 1995). Divorce can also be a non‐selected by‐product of, for example, an inability to find previous partners (Arai et al., 2009; Choudhury, 1995), or a competitor taking over the breeding position (Ens et al., 1993). Divorce is considered non‐adaptive if it is not correlated with partnership quality (e.g. reproductive output) prior to divorce and does not lead to increased partnership quality post‐divorce for at least one ex‐partner. However, it is challenging to pinpoint the exact factors driving divorce in natural populations (Culina, Radersma, et al., 2015).

Five methodological issues hinder our understanding of divorce in wild animals. First, when assessing drivers of divorce, divorcers should be compared to individuals remaining in their partnership and exclude widows, as widowhood is a passive rather than an active transition (Culina & Brouwer, 2022). Second, consequences of widowing and divorcing are often not studied simultaneously or distinguished from one another (Culina, Radersma, et al., 2015). Third, both short‐ and long‐term reproductive consequences need assessing, as divorce may have advantages that emerge after any negative impact of being in a new partnership is overcome (Naves et al., 2007). To date, we are aware of two studies investigating long‐term reproductive consequences of divorce (i.e. beyond the immediate subsequent breeding season or reproductive event), both in seabirds (Heg et al., 2003; Sun et al., 2022). Fourth, many studies do not account for the age of divorcees. As age often correlates with reproductive success and survival (Nussey et al., 2013; Ricklefs & Scheuerlein, 2001), it may mask the costs and benefits of divorcing if divorce itself is age‐dependent. Additionally, the potential to enhance future reproductive success may lead to age‐specific divorce propensities, for example, with higher divorce rates in younger individuals. Fifth, since divorce is a jointly expressed trait, divorcees are often not distinguished between initiators and victims of divorce, although the mechanism of divorcing and thus likely fitness consequences differ between groups (Heg et al., 2003).

Here, we account for these limitations using a 24‐year dataset of the socially monogamous Seychelles warbler (*Acrocephalus sechellensis*). Their maximum recorded lifespan is 19 years (Hammers & Brouwer, 2017) with an average lifespan of 5.5 years (Komdeur, 1991). The island population has virtually no migration (Komdeur et al., 2004, 2016), and extremely high resighting rates (Brouwer et al., 2010) allowing us to track individuals throughout their lifetime. This enables us to (1) have accurate measures of fitness proxies, (2) identify long‐term partnerships and disentangle divorce and widowhood, (3) age all individuals and (4) account for ecological and sociological effects on partnership quality.

To test if divorce is adaptive, we first assess 10 potential drivers of divorce (Table 1). If divorce is adaptive, we expect divorcees to have lower reproductive output and lower quality partners prior to divorce compared to non‐divorcees. Divorce may also be a mechanism for inbreeding avoidance to overcome initial ‘errors’ of mate choice resulting in incestuous pairings. High pairwise relatedness and subsequent inbreeding are detrimental for Seychelles warblers (Brouwer et al., 2007), although they do not avoid inbreeding in their initial mate choice (Richardson et al., 2004; Wright et al., 2016). Second, we examine whether changes in partner quality (measured as body mass and relatedness) and territory quality post‐divorce compared to pre‐divorce differed between divorcees, non‐divorcees and widows. Here, divorcees were classified as keeping (assumed initiators) or losing the resident breeding position (assumed victims). Third, we assess short‐term reproductive consequences of divorce independent of pre‐divorcing conditions by analysing whether divorcees have higher reproductive success directly after divorcing compared to widows and non‐divorcees. Fourth, we assess the long‐term consequences of early‐life divorce (≤3 years) by comparing the long‐term (>3 years old) reproduction and survival of divorcees (assumed initiators and victims) to non‐divorcees. Positive effects of divorce on partner/territory quality, reproductive success, and survival compared to widowing/not divorcing suggest that divorce is an adaptive mate‐switching strategy. Altogether, this provides a comprehensive analysis of what drives divorce in a natural vertebrate system, and whether there are any costs associated with mate switching.

**TABLE 1:** Potential causes of divorce in the Seychelles warbler, how they are measured and the predicted effect they will have on divorce propensity based on earlier findings in Seychelles warblers and other socially monogamous species.

	Parameter	Estimation	Predicted effect on divorce
1	Clutch size	Number of eggs produced in the first clutch of the breeding season (including any eggs by co‐breeding subordinate females)	Divorce propensity decreases with clutch size, since a large clutch is a cue of a high‐quality partnership (meta‐analysis; Culina, Radersma, et al., 2015)
2	Genetic offspring of female	Number of fledged genetic offspring produced by the female per breeding season	Individuals producing more genetic offspring are less likely to divorce, since this can be a cue of a high‐quality partnership
3	Age	The age of the male and/or female in the field season	Young individuals divorce more due to lack of breeding experience, poorer ability to retain their territory and/or partner and/or (conversely if high quality) have more energy to allocate to mate searching (Pyle et al., 2001). Very old individuals also divorce more, Seychelles warblers senesce (Hammers et al., 2015) and may, therefore, be more likely to be outcompeted or divorced by their partner for a younger partner
4	Pair‐bond tenure	Length of partnership since the first breeding season they were pair‐bonded	Partnerships that last longer are less prone to divorce, since these are likely high‐quality pair bonds (as they did not divorce before), and due to pair familiarity and improved coordination (Culina et al., 2020; Sánchez‐Macouzet et al., 2014; van de Pol et al., 2006)
5	Pairwise relatedness	Genetic relatedness between pair‐bonded individuals	In the Seychelles warbler, inbreeding – as a measure of multilocus homozygosity – is negatively associated with fitness proxies, as maternal homozygosity negatively affects offspring survival (Brouwer et al., 2007), and homozygosity is negatively correlated with telomere length (Bebbington et al., 2016)—a biomarker that reflects somatic condition and ultimately survival in the Seychelles warbler (Barrett et al., 2013). Although Seychelles warblers do not avoid inbreeding in their initial mate choice (Richardson et al., 2004; Wright et al., 2016), divorce may be a mechanism to escape partnerships with high pairwise relatedness (Cockburn et al., 2003; Hidalgo Aranzamendi et al., 2016), particularly in territorial cooperatively breeding species with highly non‐random spatial distribution of relatedness among individuals (Hatchwell et al., 2000).
6	Body mass	Body mass of individual in the given field season	Individuals are more likely to divorce partners with lower body mass (meta‐analysis; Jeschke & Kokko, 2008). Body mass reflects physiological condition in the Seychelles warbler, and individuals in better environments have higher body mass (Bebbington, Kingma, Fairfield, Dugdale, et al., 2017; Bebbington, Kingma, Fairfield, Spurgin, et al., 2017; Brouwer et al., 2009)
7	Territory quality	Invertebrate prey availability, linked to territory size, vegetation and prey abundance (Brouwer et al., 2009; Komdeur, 1992)	Pairs in poor‐quality territories are more likely to divorce to escape poor breeding conditions (reviews; Black, 1996; Cézilly et al., 2000)
8	Population density	The number of adults in the population representing competition for a breeding vacancy	When population density is very high, individuals may be less likely to divorce due to competition involved in finding a new breeding position (Komdeur & Edelaar, 2001) Divorce is more likely when population density is high, since there are more potential partners and mating opportunities (review; Kokko & Rankin, 2006)
9	Population adult sex ratio	Ratio of adult males/females in the population, which represents the level of competition for a breeding vacancy and/or scarcity of potential partners per sex	Individuals are more likely to divorce when the sex ratio is unbalanced and there is a rare sex (Liker et al., 2014) Natal dispersal propensity is driven by adult sex ratio in the Seychelles warbler (Speelman et al., 2024); thus, divorce may be a similar strategy in response to adult sex ratio variation
10	Helper presence	Presence of (1) male and (2) female helpers (Y/N)	Helpers alleviate workload for breeders in the Seychelles warbler (van Boheemen et al., 2019), thereby breeders may be less likely to divorce and leave the territory. Helpers may also reflect previous successful reproduction as they are often retained offspring (Groenewoud et al., 2018). Male and female helper presence affect divorce propensity differently, as females are more likely to help (Hammers et al., 2019) and help more (Richardson, Burke, et al., 2003)

*Note*: Potential causes include indicators of pair‐bond and partner quality (1–6), and socio‐ecological factors (7–10).

## METHODS

The Seychelles warbler is a passerine endemic to the Seychelles archipelago. The study population on Cousin Island (29 ha, 04°20′ S, 55°40′ E) typically consists of ~320 birds distributed across ~115 territories (Hammers et al., 2015; Speelman et al., 2024). Seychelles warblers are facultative cooperative breeders, with approximately 50% of territories containing mature subordinates (Kingma et al., 2016). For each major (June–September) and minor (January–March) breeding season, all individuals are tracked to assign territory residency, social status, and territory boundaries (Bebbington, Kingma, Fairfield, Dugdale, et al., 2017). Dominant breeders are identified through pair and courtship behaviours, and helpers and non‐helping subordinates through brooding and nestling provisioning (Komdeur, 1992; Richardson et al., 2002; Richardson, Komdeur, et al., 2003; van Boheemen et al., 2019). Resighting rates are very high (98% ± 1% SE; Brouwer et al., 2006, 2010), meaning that individual survival can be accurately assessed. Since Seychelles warblers predominantly feed on insects on the undersides of leaves (Komdeur, 1991), territory quality is measured using mean monthly insect density corrected for the plant species present (Brouwer et al., 2009; Komdeur, 1992). Population density was measured using all adults (dominants and subordinates) known to be alive during a breeding season. Adult sex ratio (ASR) was the proportion of male adults.

Each season as many individuals as possible are caught using mist nets, blood‐sampled (~25 μL) and their body mass measured. If caught for the first time, individuals are banded with a unique metal and three colour rings. First‐caught individuals are aged based on hatch day, or behaviour and eye colour (Brown et al., 2022). DNA extracted from blood is used for sex determination (Sparks et al., 2022), parentage assignment (Sparks et al., 2022), and relatedness estimation (Brouwer et al., 2007; see Supplementary Methods). Seasonal reproductive success is quantified using the clutch size (which could include eggs laid by subordinate females and extra‐pair offspring; Richardson et al., 2001, 2002) and genetic offspring that reached at least 3 months of age (see Supplementary Methods).

### Data selection

We used all social breeding partnerships starting between 1995 and 2021 (*N* = 1333). Partnerships ended by translocation of one partner to another island for conservation reasons (Richardson et al., 2006; Wright et al., 2014) were categorized as ‘forced widowhood’ (*N* = 48). Divorce was defined as pair‐bond disruption in a breeding season where both partners were alive but at least one partner lost breeding status in their territory. We excluded 22 uncertain divorces due to inconsistencies in the observational data as well as possible temporary divorce events, whereby partners separated for one breeding season and reunited the next.

Information on morphometrics and territory quality was not available for every individual during every season. While individuals are caught repeatedly during their lives, not all individuals are caught each breeding season. If the individual was not captured and sampled during a breeding season, body mass measures were extrapolated from the nearest measurement in time (with a maximum of two breeding seasons, i.e., 1 year) from sexual maturity at 9 months old onwards (Komdeur, 1991). Territory quality measures were extracted from the nearest season, of the same season type, following Raj Pant et al. (2019). The total dataset with extrapolations from within 1 year (*N* = 3677) increased the sample size and power of our dataset and enabled models to converge (we had a complete measurement dataset of *N* = 253 without extrapolations, and a total of 549 female mass, 722 male mass and 2045 territory‐quality measures), although this may introduce some bias in the dataset due to cross‐seasonal variation in body mass and territory quality.

To analyse how divorce affected long‐term breeding success (>3 years old), we took a subset of our data including only early‐life divorces (within the first 3 years of life) as well as individuals that never divorced in their lifetime. As reproductive performance depends on age, reproductive costs and benefits of divorce are likely age specific and covary with pair‐bond tenure. Reproductive output changes nonlinearly with age in the Seychelles warbler (Hammers et al., 2012; Raj Pant et al., 2020), and the long‐term impact of divorce is likely age dependent and potentially masked if senescent decline in reproduction and age‐dependent likelihood of divorce per se is related to lowered long‐term reproduction after divorce. Most divorces (62%, 93/149) occur within these first 3 years of life, when reproductive success is increasing with age.

### Statistical analyses

Analyses were performed using R 4.3.0 (R Core Team, 2023). Generalized linear mixed models (GLMMs) and linear mixed models (LMMs) were fitted using *lme4* 1.1.29 (Bates et al., 2015) using the *Bobyqa* nonlinear optimization. We did not include individual breeding experience since this was collinear with age. Continuous predictors were standardized (mean centred and divided by 1*σ*) and predictors were checked for collinearity (all VIF <3). We checked models for under‐ or over‐dispersion, and residual spatial and temporal autocorrelation using *DHARMa* 0.4.5 (Hartig, 2022) and found none. For all analyses, sex‐specific models were run to avoid model overparameterization, and because males are more variable than females in their reproductive success (Sparks et al., 2022).

### Causes of divorcing

We tested our hypotheses on the causes of divorce (Table 1) using model averaging. We created a global binomial GLMM (divorce/non‐divorce) with a ‘cloglog’ link, using fixed effects for the predictors from Table 1. We added the type of breeding season (major/minor) as a fixed effect to control for seasonal differences in reproduction likelihood (Komdeur & Daan, 2005). Reproductive output is age dependent, increasing initially and then declining after ~6 years old (Hammers et al., 2012; Raj Pant et al., 2020); therefore, we included linear and squared age terms, and their interactions with breeding success measures. We also added an interaction between population density and ASR, as their effects on breeding opportunities are interdependent (Speelman et al., 2024). As random effects, we included male, female, field season and territory identity. We built competing models from the global GLMMs using the *dredge* function in *MuMIn* 1.46.0 (Bartoń, 2022) and selected plausible models using ΔAICc ≤7 (Burnham et al., 2011). We report full averages of all model set parameters, that is, setting the predictor estimate and variance to zero when a variable is absent from the model which is recommended for studies aiming to determine the relative importance of multiple factors on a response variable (Grueber et al., 2011; Nakagawa & Freckleton, 2011).

### Consequences of divorcing

We first compared individuals undergoing partner change (divorcees/widows). We ran separate LMMS to test whether divorcees: (1) have a heavier partner (Δpartner mass), (2) are less related to partner (Δrelatedness) and (3) obtain better territories (Δterritory quality) post‐ compared to pre‐mate switching, compared to widows. Fixed effects included breeding season (major/minor) and ‘type’ of pair disruption including whether the bird kept the resident breeding position (1 = divorced and kept position; 2 = divorced and lost position; 3 = widowed and kept position; 4 = widowed and lost position; 5 = forcefully widowed and kept position). As random effects, new partner, old partner, territory and field season identity were included. For the Δterritory quality model, we only compared divorcees and widows switching resident territory, and included field season and territory identity as random effects. When categorical fixed effects with >2 levels were significant, we ran pairwise comparisons of the levels by calculating the least‐squares means using *lsmeans* 2.30.0 (Lenth, 2016).

To evaluate the reproductive consequences, we constructed competing GLMMs with a Poisson and negative binomial error structure and log link function, respectively, and found all Poisson models had the best fit (>3 ΔAIC). For short‐term reproductive consequences, the response variable was the number of genetic offspring in the following breeding season, as this fitness proxy is more reliable than social offspring since it excludes extra‐pair offspring. Fixed effects included partnership status (divorced/widowed/non‐divorced), current pair‐bond tenure, breeding season type (major/minor), age and age squared and presence of female (yes/no) and male (yes/no) helpers (see Komdeur, 1994). As the age of the breeding females and males are only weakly correlated (Sparks et al., 2022), they were included in the same model. Random effects consisted of focal individual, new partner, territory and field season identity. To test if divorce leads to increased reproductive success, divorcees should be compared to the same individuals had they not divorced in response to a failed breeding attempt. Since this is impossible to test empirically, we compared reproductive consequences of mate switching with poorly performing non‐divorcees (where the pair did not raise any offspring), to see if divorcees produced more offspring compared to poorly performing pairs that subsequently did not divorce.

Next, we analysed the impact of early‐life divorce (<3 years old) on long‐term breeding success (total genetic offspring produced after 3 years of age). Fixed effects included early‐life divorce category (i) divorced and stayed: divorcees keeping their resident territory; (ii) divorced and lost: divorcees who were demoted or dispersed and (iii) never divorced, and longevity and age of first reproduction attempt (AFR) after 3 years old to control for reproductive lifespan. Random effects included field season identity and territory of first reproduction (after 3 years). To test whether annual rather than total reproductive output was mediated by early‐life divorce, we ran separate models for annual reproductive success (see Supplementary Methods).

Finally, we ran Cox mixed effects proportional hazards models using *coxme* 2.2.17 (Therneau, 2022) to test the effect of early‐life divorce on bi‐annual survival, using early‐life divorce category and maternal age at conception (as this affects survival in Seychelles warblers; Sparks et al., 2022), as fixed effects, and mother identity and season of first reproduction attempt (after 3 years) as random effects. Year of death was defined as the first year when the individual was no longer seen, with right censoring for individuals still alive at the latest sampling date. Model assumptions were confirmed using Schoenfeld's residuals (Grambsch & Therneau, 1994).

## RESULTS

**FIGURE 1:**
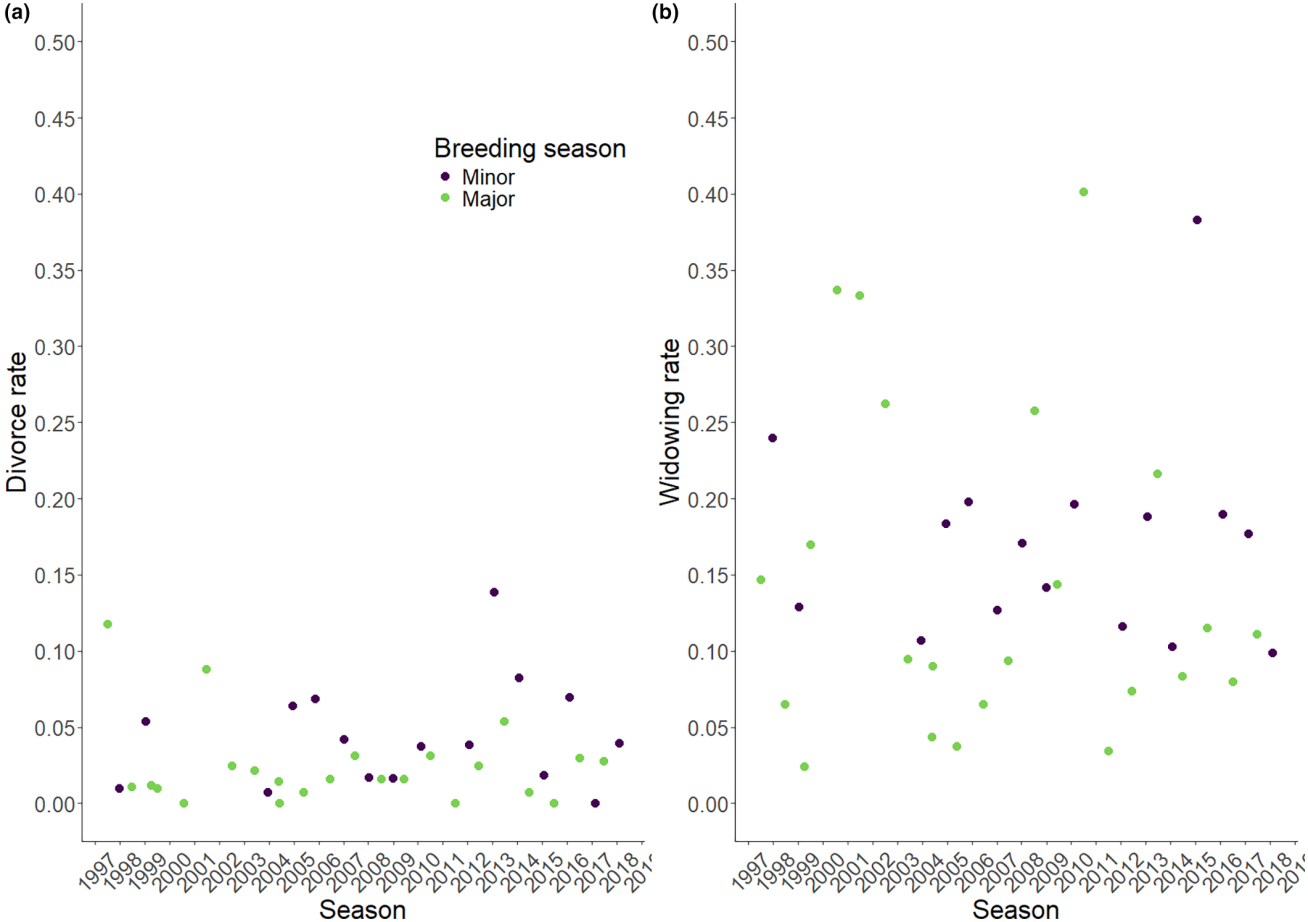
Seasonal (a) divorce rates (range 0–0.131) and (b) widowing rates (range 0.017–0.462) in the Seychelles warbler between 1997 and 2021, separated by major (purple) and minor (green) breeding seasons.

Of 1063 partnerships monitored (1997–2021), involving 621 females and 632 males, 14% ended in divorce (*N* = 149), with a mean seasonal divorce rate of 0.035 (SE = 0.005) of all pairs (*N*_seasons_ = 44), although divorce rate varied greatly over seasons (range 0–0.131; Figure 1a). Some individuals divorced twice (*N*_females_ = 14, *N*_males_ = 15) or thrice (*N*_females_ = 2, *N*_males_ = 2) in their lifetime (*N*_females_ = 12.3%, *N*_males_ = 13.0%). Sixty‐nine per cent of partnerships ended with widowhood (*N* = 733), and seasonal widowing rates also varied (mean ± SE = 0.172 ± 0.017, Figure 1b), but were similar between the sexes (females: mean ± SE = 0.09 ± 0.009; males: mean ± SE = 0.08 ± 0.008).

**FIGURE 2:**
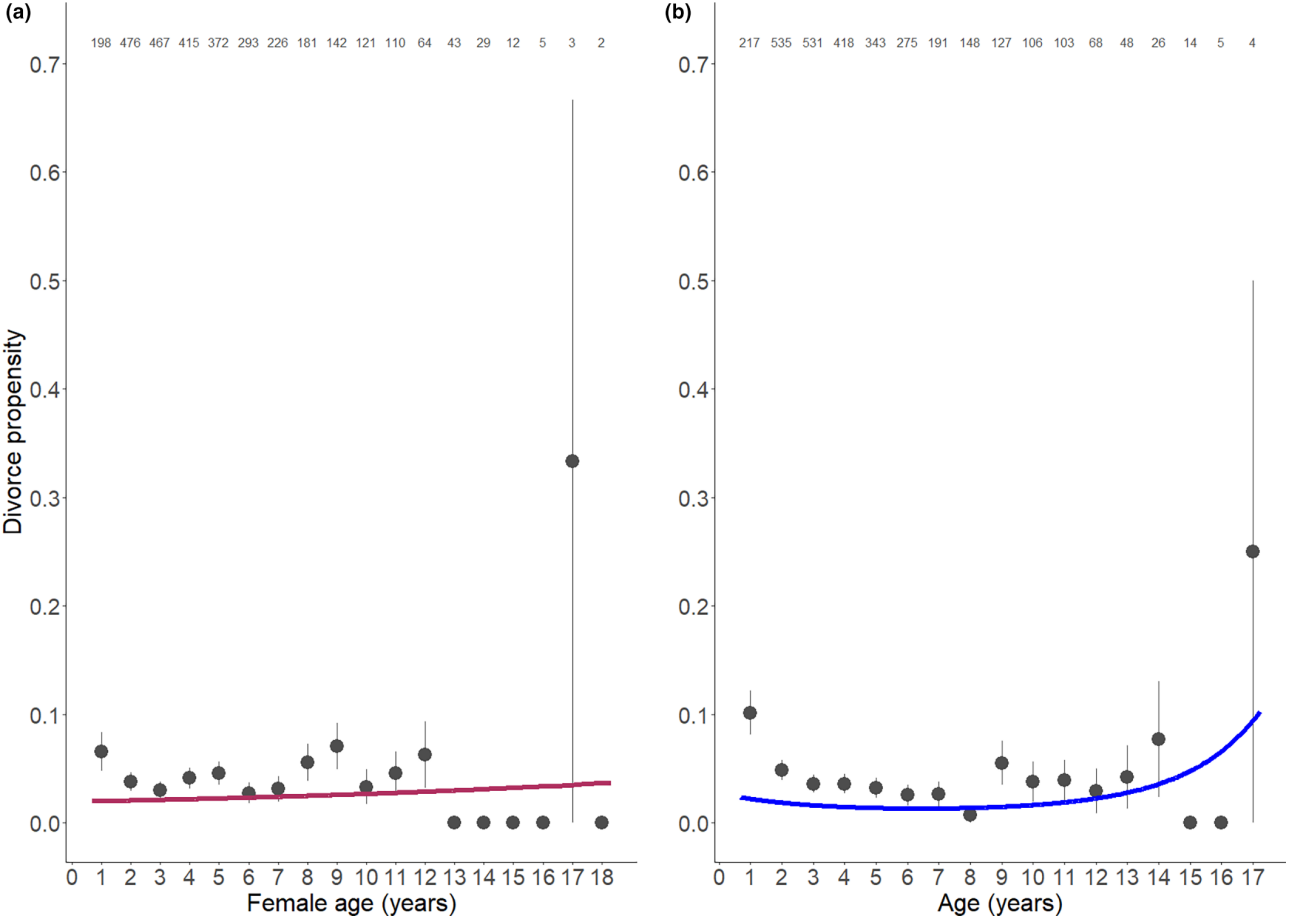
Divorce propensity in the next season in relation to (a) female and (b) male age in the Seychelles warbler between 1997 and 2021. Lines depict the model‐averaged predictions (see main text of the (a) female and (b) male trait model). Data points represent raw means and error bars the standard error of the mean. Numbers above data points represent the number of individuals.

Seasonal propensity to divorce was predicted by male age, pair‐bond tenure and clutch size (Table 2). For males, divorce exhibited a quadratic relationship with age: divorce propensity decreases with age until ~6–7 years old, and then increases again, indicating that both young and old males are more likely to divorce (Table 2; Figure 2). We fitted a linear relationship between (1) male age until the peak and (2) including and after the peak and divorce using the best‐supported model from the male traits model set, and found divorce propensity indeed decreases with male age and then increases post‐peak (Table S1). Pair‐bond tenure also predicted divorce: partners with shorter pair‐bond tenure were more likely to divorce (Table 2). Most divorces occurred in the first year of the partnership (61%, *N* = 92). Clutch size negatively predicted divorce: as more eggs were produced partners were less likely to divorce (Table 2). This effect did not vary with the age of either breeder. Of all divorces, 64% (*N* = 97) occurred when no eggs were produced. We found no effect of any other variable on divorce propensity (Table 2).

**TABLE 2:** Full model‐averaged parameter estimates of pair‐bond quality, individual quality and socio‐ecological factors on divorce propensity by the next season in the Seychelles warbler.

Fixed effects	A. Females							B. Males						
	*β*	SE	*z*	*p*	CI lower	CI upper	*N* models	*β*	SE	*z*	*p*	CI lower	CI upper	*N* models
Intercept	**−3.275**	**0.127**	**25.721**	**<0.001**	**−3.525**	**−3.026**	–	**−3.503**	**0.138**	**25.455**	**<0.001**	**−3.773**	**−3.233**	**–**
Age	0.108	0.086	1.263	0.207	−0.060	0.276	1097	**−0.163**	**0.074**	**2.197**	**0.028**	**−0.309**	**−0.018**	**2704**
Age^2^	−0.004	0.023	0.161	0.872	−0.041	0.048	578	**0.222**	**0.048**	**4.631**	**<0.001**	**0.128**	**0.316**	**2677**
Genetic offspring	−0.323	0.186	1.738	0.083	−0.687	0.041	1256	−0.209	0.148	1.413	0.158	−0.499	0.081	2021
Clutch size	**−0.523**	**0.159**	**3.292**	**0.001**	**−0.835**	**−0.212**	**1551**	**−0.515**	**0.153**	**3.359**	**<0.001**	**−0.816**	**−0.215**	**2708**
Pair tenure	**−0.462**	**0.067**	**6.862**	**<0.001**	**−0.594**	**−0.330**	**1667**	**−0.367**	**0.065**	**5.686**	**<0.001**	**−0.493**	**−0.240**	**2707**
Territory quality	0.067	0.072	0.937	0.349	−0.073	0.207	837	0.079	0.076	1.049	0.294	−0.069	0.228	1475
Body mass	−0.010	0.024	0.398	0.681	−0.057	0.038	445	−0.008	0.023	0.346	0.729	−0.053	0.037	852
Pairwise relatedness	0.138	0.226	0.610	0.542	−0.305	0.582	630	0.163	0.244	0.666	0.505	−0.316	0.641	1118
Population density	0.046	0.080	0.577	0.564	−0.111	0.204	586	0.056	0.087	0.649	0.516	−0.114	0.226	1140
Population adult sex ratio	−0.036	0.078	0.457	0.648	−0.189	0.118	558	−0.037	0.074	0.449	0.618	−0.181	0.108	1048
Female helper presence (Y)	0.027	0.107	0.255	0.799	−0.183	0.237	408	0.061	0.172	0.355	0.722	−0.277	0.399	904
Male helper presence (Y)	−0.111	0.416	0.267	0.790	−0.926	0.704	464	−0.134	0.486	0.276	0.782	−1.088	0.819	907
Breeding season (major)	−0.131	0.206	0.635	0.526	−0.534	0.273	664	−0.143	0.207	0.692	0.489	−0.548	0.262	1161
Age × Genetic offspring	0.002	0.053	0.040	0.968	−0.101	0.105	198	−0.020	0.052	0.375	0.708	−0.122	0.083	618
Age^2^ × Genetic offspring	0.001	0.015	0.062	0.951	−0.028	0.030	66	−0.001	0.026	0.022	0.982	−0.051	0.050	562
Age × Clutch size	0.010	0.035	0.279	0.780	−0.058	0.078	274	−0.075	0.114	0.655	0.512	−0.298	0.149	1073
Age^2^ × Clutch size	−0.002	0.018	0.127	0.899	−0.038	0.033	104	−0.071	0.086	0.827	0.408	−0.240	0.098	1150
Population adult sex ratio × density	−0.001	0.014	0.048	0.962	−0.029	0.027	31	−0.001	0.017	0.084	0.933	−0.034	0.031	90
Random effects	*σ* ^2^	*N*						*σ* ^2^	*N*					
Male ID	0.008	486						0.0002	486					
Female ID	0.579	465						0.522	465					
Territory ID	0.005	168						0.001	168					
Field season ID	0.813	39						0.807	39					

*Note*: Sex‐specific models were run. Candidate models = 24,320, top models = 1667 (female traits) and 2766 (male traits). Included are the model‐averaged estimates (*β*), standard errors (SE), the 95% confidence intervals (CI) of fixed effects and the total number of models (*N*) with the fixed effect in the top model set (ΔAICc ≤7). Random effect variances (*σ* ^2^) and number of levels (*N*) of the best models are reported. Reference category is no male or female helpers. Effects with a CI that does not overlap 0 are in bold.

After divorcing, 80 males (53%) and 58 females (35%) maintained their breeding position in the resident territory (Table 3). Dispersal to a breeding position in another territory was similar among the sexes (*N*_females_ = 29, 19%; *N*_males_ = 35, 23%), whereas more females (*N* = 34, 23%) than males (*N* = 9, 6%) became subordinates post‐divorce. Overall, 39% of divorcees losing their breeding position in the resident territory found a dominant breeding position elsewhere. Of 43 divorcees that became subordinates, four females and one male were a parent of the new breeder. Females (*N* = 22) ‘step down’ from their breeding position for a single breeding season whilst still remaining in their resident territory more often than males (*N* = 4). These females can be mothers of the new breeder that replaces them temporarily (*N* = 4). We did not classify these events as divorces, since these individuals stay within the same territory whilst taking a single‐season break from breeding before re‐joining their partner.

**TABLE 3:** Social status of Seychelles warblers in the breeding season after divorce or widowhood for males and females separately.

Status	Males			
	Divorced (*N* = 151)		Widowed (*N* = 346)	
	Stayed	Dispersed	Stayed	Dispersed
Breeder	80	35	320	17
Subordinate	7	2	3	1
Unknown status/residency^a^	27		5	

Status	Females			
	Divorced (*N* = 151)		Widowed (*N* = 388)	
	Stayed	Dispersed	Stayed	Dispersed
Breeder	58	29	352	16
Subordinate	26	8	4	1
Unknown status/residency^a^	30		15	

^a^ Includes Birds that are alive, but were only seen once or twice in any territory, so were either of ‘unknown’ social status or floaters.

Divorcees did not improve or deteriorate in the quality of their territory (Table S2) or partner body mass (Table S3) compared to widows and forced widows (translocated partner), nor was there a difference between the partner body mass of individuals keeping or losing their resident breeding positions. However, males that divorced and dispersed were significantly more related to their new partner (Δ*relatedness*: mean ± SE = 0.129 ± 0.294) compared to widowed males keeping their territory (Table S4). This effect disappeared when outliers of pairwise‐relatedness were removed (>2*σ* from median, *N*_removed_ = 5, Table S5). In females, there were no differences in change in pairwise relatedness (Table S6).

Short‐term breeding success was affected by helper presence, age, and breeding season but not by being a divorcee, (forced) widow or non‐divorcee even when controlling for dispersal after pair‐bond disruption (Table S7). When we compared divorcees and widows with poorly performing non‐divorcing pairs (where the female did not raise any genetic offspring in the prior season), our findings remained consistent (Table S8).

**FIGURE 3:**
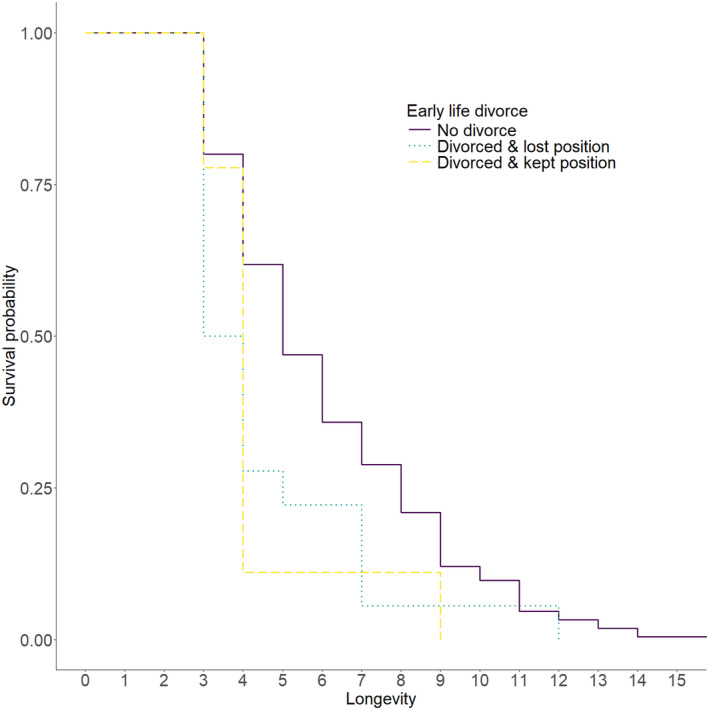
Survival probability of female Seychelles warblers in each early‐life (<3 years) divorce category (see key; *N* = 274). The data include all females who survived for at least 3 years that were included in the Cox mixed effects proportional hazards model (Table S9A).

In the longer term, we found that divorced females losing their resident breeding position in early life had lower later‐life survival than females who never divorced (Table S9A; Figure 3). However, divorced females keeping their breeding position in the resident territory did not differ in later‐life survival compared to females who divorced and did not keep their resident breeding position. We did not find an effect of early‐life divorce on later‐life annual survival post‐divorce in males (Table S9B).

Long‐term breeding success (while controlling for survival) did not depend on whether individuals divorced within the first 3 years of life and whether they kept their resident breeding position (Table S10). Long‐term breeding success is highly correlated with longevity (Pearson's correlation; *r* = 0.755, *t*_535_ = 26.643, *p* < 0.001), and when longevity was removed, the effect size of divorce on breeding success increased (Table S11). This suggests that the potential effect of divorce on long‐term breeding success is driven by longevity rather than differences in seasonal reproduction. However, the sample size of different classes related to divorce for which we had full lifetime data was limited and skewed (females: *N*_lost resident position_ = 13, *N*_kept resident position_ = 8, *N*_never divorced_ = 196; males: *N*_lost resident position_ = 10, *N*_kept resident position_ = 6, *N*_never divorced_ = 236). Likewise, seasonal reproductive success after 3 years old until death (rather than long‐term reproductive success) did not differ between early‐life divorcees (keeping the resident breeding position or not) and non‐divorcees, nor did we find an age‐dependent effect of divorce on reproduction (Table S12).

## DISCUSSION

Our study identified 1063 breeding partnerships with 149 ending in divorce and 733 in widowing in the Seychelles warbler. Partners that had recently been pair‐bonded, and those that produced fewer eggs, were more likely to divorce the following season. Both younger and older males exhibited higher divorce propensity than middle‐aged males, whereas female age did not predict divorce. We did not identify any short‐term fitness consequences of divorcing. Long‐term survival of females, but not males, divorcing early in life and losing their residential status was 2.33 times lower than females never divorcing. Early‐life divorce, when controlling for longevity, was not associated with long‐term reproductive success post 3 years of age (for those females that survived).

### Causes of divorce

Higher divorce propensity was linked with lower egg production in the preceding breeding season suggesting poor reproduction may drive divorce. Specifically, measures early in the reproductive period (clutch size) rather than later indicators (number of fledglings) predicted divorce. This is in line with previous meta‐analyses (Culina, Radersma, et al., 2015; Dubois & Cézilly, 2002) showing early breeding stages may provide a more accurate reflection of pair‐bond quality, as they are less influenced by stochastic environmental factors. In Seychelles warblers, offspring survival depends on nest guarding against predators, provisioning and helper presence (Komdeur, 1991; Komdeur & Kats, 1999) as well as stochastic effects like heavy rainfall (Borger et al., 2023). After egg laying, individuals have invested into nest building, egg production, and clutch guarding (Komdeur, 1994; Richardson, Burke, et al., 2003; Richardson, Komdeur, et al., 2003), which may provide sufficient information to decide on partnership continuation. Although clutch size is an imperfect measure of breeding success due to extra‐pair paternity (Hadfield et al., 2006) and subordinate females reproducing (Raj Pant et al., 2019; Richardson et al., 2002), it still reflects the ability to produce and protect eggs, and possibly pair‐bond quality. Theoretical models have shown that divorce can evolve as an adaptive strategy when information about the breeding scenario is imperfect or unattainable (Lerch et al., 2022; McNamara & Forslund, 1996). While increased pair‐bond familiarity over time may reduce divorce likelihood in response to failed breeding at later stages, this remains untested in Seychelles warblers.

The age of males, but not females, predicted divorce: young, inexperienced males and old, senescent males (Hammers et al., 2012) were more likely to divorce or be divorced by their partner, with the lowest divorce rate coinciding with their reproductive peak (Raj Pant et al., 2020). The higher incidence of divorce in young males may be driven by three non‐mutually exclusive mechanisms. First, young males may divorce more due to poor initial mate choice (Choudhury, 1995), although males did not improve their partner quality post‐divorce. Second, younger males may lack experience, leading to increased partner departure or expulsion by a competitive intruder (Choudhury, 1995; Ens et al., 1993). Third, investing in finding a higher quality partner early in life (even if this results in immediate costs) may increase future reproductive output (Choudhury, 1995). After the reproductive peak, males may be divorced more due to senescence, with declining reproductive output (Hammers et al., 2012; Raj Pant et al., 2020), and competitive ability. There is no strong age‐assortative mating in Seychelles warblers (Sparks et al., 2022), meaning that jointly expressed traits such as divorce can have different age‐dependencies between partners as they do not covary. Overall, the above‐mentioned hypothesised mechanisms behind the influence of age on divorce likely differ between the sexes, whereby one sex is more often initiator than victim of divorce.

Divorce likelihood decreased as partners stayed together across breeding seasons, which is consistent with empirical (e.g. Naves et al., 2007; van de Pol & Verhulst, 2006) and theoretical studies (McNamara et al., 1999; McNamara & Forslund, 1996). The ‘mate familiarity effect’ argues partner retention increases breeding performance through increased coordination and cooperation (Choudhury, 1995), especially in long‐lived species with biparental care. However, it is difficult to disentangle from individual age‐specific effects (Sánchez‐Macouzet et al., 2014) and selective disappearance of poor‐quality partnerships. In our study, the effect of pair‐bond tenure on divorce is unlikely to be driven by age or breeding experience as these are not collinear with pair‐bond tenure, and old males are more likely to divorce. While it is possible that poor‐quality partnerships disappear early whereas high‐quality pair‐bonds persist, we found no evidence for a positive relationship between pair‐bond tenure and reproduction in our 24‐year dataset. We cannot distinguish within‐ and between‐individual effects of age and pair‐bond tenure on divorce, as we have too few samples of Seychelles warblers divorcing more than once. A final explanation for reduced divorce over pair‐bond tenure is that longer lasting partnerships are better able to resist takeover attempts from competitors (Jeschke et al., 2007), possibly due to better coordinated territory and mate defence, as divorce might not be an active decision from within the pair bond (Taborsky & Taborsky, 1999).

### Consequences of divorce

A large proportion of divorced females (23%) became subordinates, compared to a smaller proportion of female widows (1%). In contrast, only 5% of divorced and 1% of widowed males became subordinates. This aligns with previous findings showing females are more often demoted from a dominant breeder position and become ‘grandparent’ helpers than males (Richardson et al., 2007). Our findings emphasize this sex difference is largely driven by divorcing individuals, whereby females may step down to become grandmother helpers. Four female and one male divorced breeder became grandparent helpers for related dominants, meaning they gained indirect fitness benefits through raising kin (Richardson et al., 2007). Four females, but not males, that ‘stepped down’ temporarily were also the parent of the breeder in that season.

We separated divorced individuals into assumed victims (kept) and victims (lost the resident breeding position) to control for fitness benefits of divorce being masked by costs faced by victims. However, we found no evidence of divorce being a successful strategy for assumed initiators to acquire higher quality mates or territories compared to widows, forced widows (from translocations) and assumed victims. Short‐term reproduction did not differ among divorcees (keeping the resident territory or not), (forced) widows and non‐divorcees, and pair‐bond tenure did not predict subsequent reproduction. Experimental disappearance of partners (forced widowhood) did not result in any costs, meaning there are no apparent reproductive costs that we could detect, in starting a new partnership for Seychelles warblers.

In long‐lived species, tracking reproductive success over multiple breeding seasons post‐divorce is crucial to disentangle short‐term costs from long‐term benefits and elucidate whether divorce is adaptive (Naves et al., 2007). In Seychelles warblers, divorce did not affect long‐term reproductive output (after controlling for survival), even when controlling for whether they kept the resident breeding position. However, the small sample size means that the power of this test was relatively low. One other study disentangled the long‐term reproductive output of different types of divorcees in Eurasian oystercatchers (*Haematopus ostralegus*; Heg et al., 2003), where divorcees leaving the original breeding position had lower future reproductive success compared to those keeping their breeding position. In Seychelles warblers, mortality risk differed between divorced females that lost their resident breeding position and female non‐divorcees, indicating a sex‐specific cost of divorcing but only when the resident breeding position is lost. This is congruent with the ‘better‐option’ hypothesis: one partner benefits from divorce, whereas the ‘abandoned’ partner suffers costs (Choudhury, 1995). Divorced females losing the breeding position may face survival costs by spending energy and time looking for a new territory or mate. Alternatively, lowered survival of these females might be linked to the causes of divorce (Jankowiak et al., 2018): some males may divorce females in poor condition which—although not detected in our study prior to divorce—results in higher mortality of these divorced females. Our finding aligns with previous studies finding survival costs associated with pair‐bond disruption (Culina, Lachish, et al., 2015; Jankowiak et al., 2018; Nicolai et al., 2012), but to our knowledge, ours is the first to find survival costs of divorce specifically.

Interspecies variation in divorce rate is often attributed to variation in longevity (Berec & Boukal, 2004) as short‐lived species have fewer future breeding opportunities than long‐lived species. We propose similar age‐dependent causes of divorce exist within species, where divorce rates decrease later in life due to fewer potential future breeding opportunities and benefits to be gained from divorcing. Two studies have examined the long‐term consequences of divorce: wandering albatross (*Diomedea exulans*) divorcees did not experience long‐term reproductive consequences (Sun et al., 2022) and divorced Eurasian oystercatchers faced a short‐term reduction but long‐term increase in reproductive performance (Heg et al., 2003). Our study differs by combining four aspects; we (i) consider age‐ and lifespan‐specific effects on timing of divorce, (ii) compare divorcees to non‐divorcees, (iii) account for active decision‐making by disentangling divorce participants and (iv) examined divorce in a closed population where survival can be separated from dispersal. Divorce may be a strategy with different outcomes and motivations per age class, especially in long‐lived species that experience a senescent decline in breeding success.

## CONCLUSION

In Seychelles warblers, divorce depends on prior reproductive output, pair‐bond tenure and male age. Divorcees appear to face sex‐ and breeding status‐specific consequences that emerge over longer timeframes; divorced females losing their resident breeding position have low annual survival, which provides to our knowledge the first evidence of survival costs of divorce rather than mate‐switching in general. Whether divorce is adaptive in Seychelles warblers remains unclear, because although divorce is related to reduced reproductive success, no positive consequences emerge even when controlling for breeding status. Divorce is relatively uncommon in Seychelles warblers (14%) compared to other species, for example, ~40% in song sparrows *Melospiza melodia* (Germain et al., 2018), which is likely due to high competition for breeding vacancies in the Seychelles warbler population (Komdeur & Pels, 2005; Speelman et al., 2024). Future research should (1) consider divorce as a product of different strategies which are not all necessarily adaptive but possibly a by‐product of stochastic events, (2) compare divorce (active mate‐switching) to both non‐divorcees and widows (passive mate‐switching), (3) not solely consider short‐term reproductive consequences and (4) examine age‐specific and longevity‐dependent causes and consequences of divorce. To verify true benefits of divorce, divorce should be compared to a proper control: when the same individual remains in the same (potentially poorly performing) partnership. This is impossible in empirical studies on wild populations that cannot alter partnership qualities. Additionally, as divorce is a jointly expressed trait, the initiator of divorce needs to be identified.

## AUTHOR CONTRIBUTIONS

FJDS and HLD conceived the study design and methodology, with input from DSR. FJDS performed the analyses, with input from HLD. FJDS wrote the manuscript, with input from HLD, DSR and JK. DSR organized (and with many others – see acknowledgments) undertook fieldwork. Molecular parentage assignment methods were developed and undertaken by DSR and HLD. HLD, DSR, JK and TB managed the long‐term Seychelles warbler study system and database including gaining the relevant funding. All authors gave final approval for submission.

### PEER REVIEW

The peer review history for this article is available at https://www.webofscience.com/api/gateway/wos/peer‐review/10.1111/ele.14471.

## Supporting information


Data S1.

Data S2.

Data S3.

Data S4.

## Data Availability

Data and code are available on Figshare: https://doi.org/10.6084/m9.figshare.25033811.v1.
