# Supplementary material for: Causes and consequences of divorce in a long‐lived socially monogamous bird
Source: Ecol Lett. 2024 Dec 31;27(12):e14471. doi: 10.1111/ele.14471 (PMC11686947; doi:10.1111/ele.14471)
Supplement: Supplementary file 1 — Data S1. [file ELE-27-0-s004.docx]

SUPPLEMENTARY MATERIAL

1. SUPPLEMENTARY METHODS

Molecular data

DNA was extracted from blood samples with a Qiagen DNeasy Blood and Tissue Kit from 2013 onwards, or modified ammonium acetate protocol for older samples, and used to determine sex using 1-3 markers (Sparks *et al.* 2022), as well as genotyping using a panel of 30 microsatellite markers (Richardson *et al.* 2004; Sparks *et al.* 2022; Spurgin *et al.* 2014). Parentage was assigned using MasterBayes 2.52 (Hadfield *et al.* 2006) and used to build a genetic pedigree (Edwards *et al.* 2018; Sparks *et al.* 2022). Pairwise relatedness between partners was calculated using the Queller and Goodnight estimation (Queller & Goodnight 1989) using the r-package *related* 0.8 (Pew *et al.* 2015), since this is the most suitable estimator using our microsatellite panel (Bebbington *et al.* 2017). This relatedness estimation reflects pedigree relatedness in the Seychelles warbler (Brouwer *et al.* 2007), and heterozygosity across the microsatellite panel reflects genome-wide heterozygosity (Bebbington *et al.* 2016).

Reproductive success measures

Reproductive effort was measured as a single measure within the dyad (clutch size) and as a sex-specific measure (genetic offspring surviving until 3 months). Many nests could not be reached, and therefore clutch sizes were inferred from nest watch observations and from the number of fledglings sighted. Therefore, it is possible some offspring died before being sighted. However, Seychelles typically produce a single egg per season (92% of cases in this study) but two- or three-egg clutches do occur (Komdeur 1991; Richardson *et al.* 2001; N=171 out of 2230 in this study), and we have a single occurrence of a four-egg clutch. The effect of inferred clutch sizes being larger than observed will be marginal, since this method of quantifying clutch size avoids overestimation of clutch size. If multiple nests were built during a breeding season (18% of cases in this study), the clutch size of the first nest was used in analyses since early measures of breeding success are likely important drivers of mate fidelity in birds (Culina *et al.* 2015). The number of genetic offspring only includes individuals that were caught, genotyped and assigned a parent with ≥0.80 probability. In the Seychelles warbler, extra-pair fertilizations by dominant males from nearby territories are common, with ~44% of all offspring being sired by a male other than the socially pair-bonded male (Hadfield *et al.* 2006; Richardson *et al.* 2001) and 11% of offspring are assigned to a female subordinate (cobreeder) in the territory (Raj Pant *et al.* 2019). Therefore, genetic offspring are a more reliable measure of ‘true’ breeding success of an individual, but does not always reflect social parentage; those offspring for which the breeder provides parental care. Accurate information for social status and genetic offspring were available for 1997-2018; thus for every analysis including a measure of genetic offspring, the last breeding seasons (2019 onwards) were excluded from the dataset.

Annual reproductive success models

The effect of early-life divorce on long-term total reproductive success may be caused by differences in annual reproductive success post early-life. We assessed seasonal reproductive success per sex (after 3 years of age until death) using the clutch size and number of genetic offspring as a response variable and included the fixed effects of age, age-squared (both sexes), breeding season (major/minor) and a categorical variable for early-life divorce (divorce & stayed: individuals that kept their breeding position in their resident territory; divorce & lost: individuals who lost their breeding position by being demoted or dispersing to a new territory, and individuals that never divorced). Because the effect of early-life divorce on current reproduction is likely dependent on chronological age, we included two-way interactions between early-life divorce and both age and age-squared. To control for between-individual effects of age (e.g. selective appearance and disappearance), we included age of first reproduction attempt (AFR) in the first partnership after divorce and longevity (i.e. age at death) as fixed effects (van de Pol & Verhulst 2006). Finally, we included territory identity, field season identity, individual identity, and partner identity as random effects. 

REFERENCES

Bebbington, K., Kingma, S.A., Fairfield, E.A., Dugdale, H.L., Komdeur, J., Spurgin, L.G., *et al.* (2017). Kinship and familiarity mitigate costs of social conflict between Seychelles warbler neighbors. *Proceedings of the National Academy of Sciences of the United States of America*, 114, E9036–E9045.

Bebbington, K., Spurgin, L.G., Fairfield, E.A., Dugdale, H.L., Komdeur, J., Burke, T., *et al.* (2016). Telomere length reveals cumulative individual and transgenerational inbreeding effects in a passerine bird. *Molecular Ecology*, 25, 2949–2960.

Brouwer, L., Komdeur, J. & Richardson, D.S. (2007). Heterozygosity-fitness correlations in a bottlenecked island species: A case study on the Seychelles warbler. *Molecular Ecology*, 16, 3134–3144.

Culina, A., Radersma, R. & Sheldon, B.C. (2015). Trading up: the fitness consequences of divorce in monogamous birds. *Biological Reviews*, 90, 1015–1034.

Edwards, H.A., Dugdale, H.L., Richardson, D.S., Komdeur, J. & Burke, T. (2018). Extra-pair parentage and personality in a cooperatively breeding bird. *Behavioral Ecology and Sociobiology*, 72.

Hadfield, J.D., Richardson, D.S. & Burke, T. (2006). Towards unbiased parentage assignment: Combining genetic, behavioural and spatial data in a Bayesian framework. *Molecular Ecology*, 15, 3715–3730.

Komdeur, J. (1991). Cooperative breeding in the Seychelles warbler. University of Cambridge.

Pew, J., Muir, P.H., Wang, J. & Frasier, T.R. (2015). related: an R package for analysing pairwise relatedness from codominant molecular markers. *Mol Ecol Resour*, 15, 557–561.

van de Pol, M. & Verhulst, S. (2006). Age‐Dependent Traits: A New Statistical Model to Separate Within‐ and Between‐Individual Effects. *The American Naturalist*, 167, 766–773.

Queller, D.C. & Goodnight, K.F. (1989). Estimating Relatedness Using Genetic Markers. *Evolution*, 43, 258–275.

Raj Pant, S., Komdeur, J., Burke, T.A., Dugdale, H.L. & Richardson, D.S. (2019). Socio-ecological conditions and female infidelity in the Seychelles warbler. *Behavioral Ecology*, 30, 1254–1264.

Richardson, D.S., Jury, F.L., Blaakmeer, K., Komdeur, J. & Burke, T. (2001). Parentage assignment and extra-group paternity in a cooperative breeder: The Seychelles warbler (Acrocephalus sechellensis). *Molecular Ecology*, 10, 2263–2273.

Richardson, D.S., Komdeur, J. & Burke, T. (2004). Inbreeding in the Seychelles Warbler: Environment-Dependent Maternal Effects. *Evolution*, 58, 2037–2048.

Sparks, A.M., Hammers, M., Komdeur, J., Burke, T., Richardson, D.S. & Dugdale, H.L. (2022). Sex‐dependent effects of parental age on offspring fitness in a cooperatively breeding bird. *Evolution Letters*, 6, 438–449.

Spurgin, L.G., Wright, D.J., van der Velde, M., Collar, N.J., Komdeur, J., Burke, T., *et al.* (2014). Museum DNA reveals the demographic history of the endangered Seychelles warbler. *Evolutionary Applications*, 7, 1134–1143.

1. SUPPLEMENTARY TABLES

Table S1. Output of the binomial Generalised Linear Mixed Model assessing whether male age linearly predicts divorce propensity by the next season up to and including the peak (<8 years old, *N*=2616) and from the peak onwards (>6 years old, *N*=895) in the Seychelles warbler. Model structure is based on the best supported model from the male traits model set investigating the causes of divorce, where divorce quadratically predicted by male age, being lowest at ~6-7 years old. Included are the model averaged estimates (β), standard errors (SE), *z*-value, and *p*-value of fixed effects. Random effect variances (σ^2^) and number of levels (N) are reported. Effects with *p*<0.05 are in bold.

|  | Up to & including peak (<8 years) males | | | | Peak onwards (>6 years) males | | | |
| --- | --- | --- | --- | --- | --- | --- | --- | --- |
| Fixed effect | Estimate | SE | z | p | Estimate | SE | z | p |
| Intercept | **-3.360** | **0.247** | **-13.611** | **<0.001** | **-4.262** | **0.645** | **-6.607** | **<0.001** |
| Male age | **-0.339** | **0.142** | **-2.398** | **0.017** | **0.513** | **0.243** | **2.108** | **0.035** |
| Male genetic offspring | -0.234 | 0.206 | -1.134 | 0.257 | -0.613 | 0.503 | -1.220 | 0.223 |
| Clutch size | **-0.471** | **0.228** | **-2.069** | **0.039** | -0.976 | 0.552 | -1.769 | 0.077 |
| Pair tenure | -0.204 | 0.170 | -1.198 | 0.231 | -0.415 | 0.263 | -1.579 | 0.114 |
| Territory quality | 0.142 | 0.101 | 1.409 | 0.159 | 0.267 | 0.184 | 1.452 | 0.147 |
| Random effect | σ^2^ | N |  |  | σ^2^ | N |  |  |
| Male ID | <0.0001 | 477 |  |  | 1.319 | 144 |  |  |
| Female ID | 0.531 | 434 |  |  | 0.852 | 190 |  |  |
| Territory ID | <0.0001 | 168 |  |  | <0.0001 | 111 |  |  |
| Field season ID | 0.752 | 39 |  |  | 0.921 | 39 |  |  |

Table S2. Output of the Linear Mixed Model (LMM) regressing *Δterritory quality* over ‘type’ of partnership switch of individuals that did not keep the resident breeding position for females (N=18) and males (N=20) switching partners. Included are the model estimates (β), standard errors (SE), t-value (t), and the p-value (p) of fixed effects, and the random effect variances (σ^2^) and number of levels (N). Reference category of partnership switch is divorced and did not keep position, and breeding season is minor. *The number of territories was equal to the number of observations of females switching partners, so we could not calculate the random effect variance explained by territory ID for females switching partners.

|  | Females switching partners | | | | Males switching partners | | | |
| --- | --- | --- | --- | --- | --- | --- | --- | --- |
| Fixed effects | β | SE | *t* | *p* | β | SE | t | p |
| (Intercept) | 0.356 | 0.587 | -0.607 | 0.557 | 0.096 | 0.391 | 0.245 | 0.813 |
| Partnership switch: Widowed & lost position | 0.553 | 0.553 | 0.999 | 0.374 | -0.472 | 0.259 | -1.825 | 0.090 |
| Breeding season (major) | 0.709 | 0.608 | 1.166 | 0.276 | 0.472 | 0.459 | 1.029 | 0.335 |
| Random effects | σ^2^ | N |  |  | σ^2^ | N |  |  |
| Field season ID | 0.295 | 11 |  |  | 0.574 | 10 |  |  |
| Territory ID | NA* | NA |  |  | <0.0001 | 18 |  |  |
| Residual | 0.992 |  |  |  | 0.416 |  |  |  |

Table S3. Output of the Linear Mixed Model (LMM) regressing *Δmass* of partner over ‘type’ of partnership switch including whether they kept the resident breeding position (widowed and kept position, widowed and did not keep position, divorcing and did not keep position, or forcefully widowed and kept position) for females (*N*=131) and males (*N*=112) switching partners. Included are the model estimates (β), standard errors (SE), t-value (t), and the p-value (p) of fixed effects, and the random effect variances (σ^2^) and number of levels (N). Reference category of partnership switch is (1) divorced and kept position, and breeding season is minor.

|  | | Females switching partners | | | | Males switching partners | | | |
| --- | --- | --- | --- | --- | --- | --- | --- | --- | --- |
| Fixed effects | | β | SE | *t* | *p* | β | SE | t | p |
| (Intercept) | | 0.328 | 0.401 | 0.819 | 0.415 | -0.311 | 0.536 | -0.580 | 0.563 |
| Partnership switch | (2) Divorced & lost position | -0.490 | 0.492 | -0.997 | 0.321 | 0.655 | 0.657 | 0.996 | 0.321 |
|  | (3) Widowed & kept position | 0.147 | 0.390 | 0.378 | 0.859 | -0.009 | 0.561 | -0.015 | 0.988 |
|  | (4) Widowed & lost position | -0.105 | 0.590 | -0.178 | 0.706 | 0.285 | 0.703 | 0.406 | 0.686 |
|  | (5) Forcefully widowed | 0.353 | 0.638 | 0.553 | 0.583 | 0.625 | 0.804 | 0.778 | 0.438 |
| Breeding season (major) | | -0.174 | 0.314 | -0.555 | 0.583 | -0.320 | 0.312 | -1.026 | 0.307 |
| Random effects | |  | Variance | σ^2^ | N |  | Variance | σ^2^ | N |
|  | | Male_t_ ID | 0.894 | 0.945 | 131 | Female_t_ ID | 0.923 | 0.961 | 109 |
|  | | Male_t+1_ ID | 1.073 | 1.0364 | 117 | Female_t+1_ ID | 0.718 | 0.848 | 98 |
|  | | Territory ID | <0.0001 | <0.0001 | 83 | Territory ID | <0.0001 | <0.0001 | 68 |
|  | | Field season ID | 0.307 | 0.554 | 38 | Field season ID | <0.0001 | <0.0001 | 39 |
|  | | Residual | <0.0001 | 0.0003 |  | Residual | 0.741 | 0.8644 |  |

Table S4. Output of the Linear Mixed Model (LMM) regressing *Δrelatedness* over ‘type’ of partnership switch including whether they kept the resident breeding position (widowed and kept position, widowed and did not keep position, divorcing and dispersing, divorcing and did not keep position, or forcefully widowed and kept position) and pairwise comparisons for males switching partners (*N*=140). Included are the model estimates (β), standard errors (SE), t-value (t), and the 95% confidence intervals (CI) of fixed effects, and the random effect variances (σ^2^) and number of levels (N). Reference category of partnership switch is (1) divorced and kept position, and breeding season is minor. . Effects with *p*<0.05 are in bold.

|  | | Males switching partners | | | |
| --- | --- | --- | --- | --- | --- |
| Fixed effects | | β | SE | t | p |
| (Intercept) | | 0.036 | 0.061 | 0.587 | 0.558 |
| Partnership switch | (2) Divorced & lost position | 0.087 | 0.077 | 1.133 | 0.260 |
|  | (3) Widowed & kept position | -0.096 | 0.067 | -1.450 | 0.149 |
|  | (4) Widowed & lost position | -0.038 | 0.090 | -0.425 | 0.672 |
|  | (5) Forcefully widowed | -0.038 | 0.188 | -0.205 | 0.838 |
| Breeding season (major) | | 0.021 | 0.042 | 0.494 | 0.622 |
|  | |  | σ^2^ | *N* |  |
| Random effects | | Old partner ID | 0.226 | 134 |  |
|  | | New partner ID | 0.108 | 127 |  |
|  | | Territory ID | <0.0001 | 82 |  |
|  | | Field season ID | <0.0001 | 38 |  |
|  | | Residual | <0.001 |  |  |
| Pairwise comparison | | β | SE | t | p |
| (1) Divorced & kept position -  (2) Divorced & lost position | | -0.087 | 0.084 | -1.041 | 0.836 |
| (1) Divorced & kept position -  (3) Widowed & kept position | | 0.096 | 0.070 | 1.376 | 0.644 |
| (1) Divorced & kept position -  (4) Widowed & lost position | | 0.038 | 0.098 | 0.388 | 0.995 |
| (1) Divorced & kept position -  (5) Forcefully widowed | | 0.038 | 0.199 | 0.193 | 0.999 |
| **(2) Divorced & lost position -**  **(3) Widowed & kept position** | | **0.184** | **0.061** | **3.026** | **0.036** |
| (2) Divorced & lost position -  (4) Widowed & lost position | | -0.125 | 0.086 | -1.454 | 0.603 |
| (2) Divorced & lost position -  (5) Forcefully widowed | | 0.126 | 0.196 | 0.642 | 0.968 |
| (3) Widowed & kept position -  (4) Widowed & lost position | | 0.058 | 0.080 | 0.724 | 0.950 |
| (3) Widowed & kept position -  (5) Forcefully widowed | | -0.058 | 0.188 | -0.308 | 0.998 |
| (4) Widowed & lost position -  (5) Forcefully widowed | | 0.0002 | 0.202 | 0.001 | 0.999 |

Table S5. Output of the Linear Mixed Model (LMM) regressing *Δrelatedness* with partner over ‘type’ of partnership switch including whether they kept the resident breeding position (widowed and kept position, widowed and did not keep position, divorcing and dispersing, divorcing and did not keep position, or forcefully widowed and kept position) for males switching partners (*N* = 135) without outliers (>2*σ* from median). Included are the model estimates (β), standard errors (SE), t-value (t), and the 95% confidence intervals (CI) of fixed effects, and the random effect variances (σ^2^) and number of levels (N). Reference category of partnership switch is divorced kept position, and breeding season is minor.

|  | Males switching partners | | | |
| --- | --- | --- | --- | --- |
| Fixed effects | β | SE | t | p |
| (Intercept) | 0.003 | 0.051 | 0.059 | 0.953 |
| (2) Divorced & lost position | 0.096 | 0.067 | 1.431 | 0.156 |
| (3) Widowed & kept position | -0.035 | 0.055 | -0.636 | 0.526 |
| (4) Widowed & lost position | 0.068 | 0.080 | 0.059 | 0.953 |
| (5) Forcefully widowed | -0.001 | 0.148 | -0.003 | 0.997 |
| Breeding season (major) | -0.002 | 0.036 | -0.053 | 0.958 |
|  |  | σ^2^ | *N* |  |
| Random effects | Old partner ID | 0.157 | 131 |  |
|  | New partner ID | 0.129 | 121 |  |
|  | Territory ID | <0.0001 | 79 |  |
|  | Season ID | <0.0001 | 37 |  |
|  | Residual | <0.001 |  |  |
| Pairwise comparison | β | SE | t | p |
| (1) Divorced & kept position -  (2) Divorced & lost position | -0.097 | 0.073 | -1.316 | 0.682 |
| (1) Divorced & kept position -  (3) Widowed & kept position | 0.035 | 0.059 | 0.601 | 0.975 |
| (1) Divorced & kept position -  (4) Widowed & lost position | -0.068 | 0.085 | -0.799 | 0.931 |
| (1) Divorced & kept position -  (5) Forcefully widowed | 0.001 | 0.160 | 0.003 | 0.999 |
| (2) Divorced & lost position -  (3) Widowed & kept position | 0.132 | 0.055 | 2.392 | 0.129 |
| (2) Divorced & lost position -  (4) Widowed & lost position | -0.029 | 0.079 | -0.361 | 0.996 |
| (2) Divorced & lost position -  (5) Forcefully widowed | 0.097 | 0.159 | 0.611 | 0.973 |
| (3) Widowed & kept position -  (4) Widowed & lost position | 0.103 | 0.070 | 1.470 | 0.585 |
| (3) Widowed & kept position -  (5) Forcefully widowed | -0.035 | 0.151 | 0.611 | 0.973 |
| (4) Widowed & lost position -  (5) Forcefully widowed | 0.068 | 0.164 | 0.418 | 0.994 |

Table S6. Output of the Linear Mixed Model (LMM) regressing *Δrelatedness* with partner over ‘type’ of partnership switch including whether they kept the resident breeding position (widowed and kept position, widowed and did not keep position, divorcing and dispersing, divorcing and did not keep position, or forcefully widowed and kept position) for females switching partners (*N* = 180).  Included are the model estimates (β), standard errors (SE), t-value (t), and the 95% confidence intervals (CI) of fixed effects, and the random effect variances (σ^2^) and number of levels (N). Reference category of partnership switch is (1) divorced and kept position, and breeding season is minor.

|  | | Females switching partners | | | |
| --- | --- | --- | --- | --- | --- |
| Fixed effects | | β | SE | t | p |
| (Intercept) | | -0.014 | 0.059 | 0.237 | 0.813 |
| Partnership switch | (2) Divorced & lost position | 0.087 | 0.082 | 1.063 | 0.290 |
|  | (3) Widowed & kept position | 0.037 | 0.058 | 0.640 | 0.523 |
|  | (4) Widowed & lost position | 0.028 | 0.093 | 0.302 | 0.764 |
|  | (5) Forcefully widowed | -0115 | 0.122 | -0.945 | 0.390 |
| Breeding season (major) | | 0.022 | 0.036 | 0.567 | 0.576 |
|  | |  | σ^2^ | *N* |  |
| Random effects | | Old partner ID | 0.139 | 173 |  |
|  | | New partner ID | 0.129 | 157 |  |
|  | | Territory ID | <0.0001 | 94 |  |
|  | | Season ID | 0.044 | 40 |  |
|  | | Residual | 0.096 |  |  |

Table S7. Output of the Generalized Linear Mixed Model (GLMM) regressing the number of genetic offspring of males *N_obs_*=3569 and females *N_obs_*=4097 separately the season directly after potential mate switching.  ‘Type’ of partnership included whether they kept the resident breeding position (widowed and kept position, widowed and did not keep position, divorcing and dispersing, divorcing and did not keep position, or forcefully widowed and kept position) and faithful (non-divorcing) partnerships. Included are the estimates (β), standard errors (SE), *Z*-value and *p*-value of fixed effects, as well as the variance and standard deviation (σ^2^) and number of levels (N) of random effects. Effects with a *p* < 0.05 are in bold. Reference category of partnership type is ‘stayed faithful’, no presence of male and female helpers, and breeding season is minor. *No widowed males lost their resident breeding position the season following widowhood.

|  | | Females | | | | Males | | | |
| --- | --- | --- | --- | --- | --- | --- | --- | --- | --- |
| Fixed effects | | β | SE | Z | P | Estimate | SE | Z | P |
| (Intercept) | | **-1.915** | **0.211** | **-9.078** | **<0.0001** | **-1.945** | **0.211** | **-9.217** | **<0.0001** |
| Partnership type | Divorced & kept position | -0.055 | 0.237 | -0.234 | 0.815 | -0.381 | 0.348 | -1.097 | 0.273 |
|  | Divorced & lost position | -0.443 | 0.322 | -1.375 | 0.169 | -0.310 | 0.203 | -1.526 | 0.127 |
|  | Widowed & kept position | -0.173 | 0.113 | -1.526 | 0.127 | -0.796 | 0.431 | -1.157 | 0.247 |
|  | Widowed & lost position | 0.168 | 0.362 | 0.463 | 0.643 | NA* | NA | NA | NA |
|  | Forcefully widowed | 0.093 | 0.459 | 0.202 | 0.840 | -0.550 | 0.475 | -1.157 | 0.247 |
| Female age | | -0.052 | 0.045 | -1.163 | 0.245 | **0.304** | **0.056** | **5.422** | **<0.0001** |
| Female age^2^ | | **-0.147** | **0.030** | **-4.939** | **<0.001** | **-0.108** | **0.052** | **-2.086** | **0.037** |
| Male age | | **0.131** | **0.053** | **2.469** | **0.014** | **0.304** | **0.056** | **5.422** | **<0.0001** |
| Male age^2^ | | **-0.091** | **0.027** | **-3.327** | **0.001** | **-0.179** | **0.029** | **-6.283** | **<0.0001** |
| Female helper (Y) | | 0.101 | 0.084 | 1.202 | 0.229 | **0.226** | **0.088** | **2.569** | **0.010** |
| Male helper (Y) | | **0.470** | **0.108** | **4.362** | **<0.001** | **0.342** | **0.115** | **2.965** | **0.003** |
| Pair tenure | | 0.034 | 0.049 | 0.683 | 0.495 | 0.022 | 0.021 | 1.030 | 0.303 |
| Breeding season (major) | | **1.063** | **0.264** | **4.022** | **<0.001** | **1.039** | **0.252** | **4.117** | **<0.0001** |
| Random effects | | σ^2^ | N |  |  | σ^2^ | N |  |  |
| Male ID | | 0.066 | 544 |  |  | 0.375 | 499 |  |  |
| Female ID | | 0.296 | 475 |  |  | 0.388 | 482 |  |  |
| Territory ID | | 0.147 | 170 |  |  | <0.0001 | 170 |  |  |
| Field season ID | | 0.769 | 39 |  |  | 0.734 | 39 |  |  |

Table S8. Output of the Generalized Linear Mixed Model (GLMM) regressing clutch size over ‘type’ of partnership type (widowing and divorcing, keeping resident breeding position or not, respectively) and poorly performing faithful partnerships (i.e. partnerships that fail to sire offspring) for females (*N_obs_*=2115) and males (*N_obs_*=1839). Included are the estimates (β), standard errors (SE), *Z*-value and *p*-value of fixed effects, as well as the variance and standard deviation (σ^2^) and number of levels (N) of random effects. Effects with a p < 0.05 are in bold. Reference category of partnership type is ‘stayed faithful’, no presence of male and female helpers, and breeding season is minor. *No widowed males dispersed the season following widowhood.

|  | | Females | | | | Males | | | |
| --- | --- | --- | --- | --- | --- | --- | --- | --- | --- |
| Fixed effects | | β | SE | Z | P | Estimate | SE | Z | P |
| (Intercept) | | **-1.983** | **0.274** | **-7.234** | **<0.0001** | **-1.711** | **0.268** | **-6.384** | **<0.0001** |
| Partnership type | Divorced & kept position | 0.169 | 0.245 | 0.690 | 0.490 | -0.139 | 0.212 | -0.659 | 0.510 |
|  | Divorced & lost position | -0.266 | 0.325 | -0.818 | 0.413 | -0.544 | 0.359 | -1.516 | 0.130 |
|  | Widowed & kept position | -0.076 | 0.131 | -0.576 | 0.565 | -0.980 | 0.438 | -0.571 | 0.568 |
|  | Widowed & lost position | 0.228 | 0.365 | 0.624 | 0.533 | NA* | NA | NA | NA |
|  | Forcefully widowed | 0.294 | 0.476 | 0.618 | 0.536 | -0.277 | 0.485 | -0.571 | 0.568 |
| Female age | | 0.019 | 0.058 | 0.326 | 0.746 | 0.004 | 0.067 | 0.064 | 0.949 |
| Female age^2^ | | **-0.224** | **0.044** | **-5.088** | **<0.0001** | **-0.078** | **0.039** | **-1.987** | **0.047** |
| Male age | | **0.172** | **0.077** | **2.228** | **0.026** | **0.317** | **0.072** | **4.376** | **<0.0001** |
| Male age^2^ | | **-0.106** | **0.039** | **-2.701** | **0.007** | **-0.179** | **0.039** | **-4.568** | **<0.0001** |
| Female helper (Y) | | 0.222 | 0.118 | 1.884 | 0.060 | 0.361 | 0.116 | 3.108 | 0.002 |
| Male helper (Y) | | **0.377** | **0.163** | **2.309** | **0.021** | 0.092 | 0.178 | 0.520 | 0.603 |
| Pair tenure | | -0.016 | 0.028 | -0.568 | 0.570 | 0.003 | 0.026 | 0.120 | 0.904 |
| Breeding season (major) | | **1.076** | **0.305** | **3.530** | **<0.0001** | **0.802** | **0.301** | **2.669** | **0.008** |
| Random effects | | σ^2^ | N |  |  | σ^2^ | N |  |  |
| Male ID | | <0.0001 | 507 |  |  | 0.214 | 430 |  |  |
| Female ID | | 0.198 | 431 |  |  | 0.353 | 427 |  |  |
| Territory ID | | 0.125 | 165 |  |  | <0.0001 | 164 |  |  |
| Field season ID | | 0.761 | 39 |  |  | 0.760 | 39 |  |  |

Table S9. Time-dependent Cox Regression model testing the effects of early-life divorce and whether (A) females (*N*=274) and (B) males (*N*=309) kept their resident breeding position or not on bi-annual survival (2 breeding seasons per year) in the Seychelles warbler. Included are the coefficients (hazard rate), standard errors (SE), HR (hazard ratio), *Z*-value and *p*-value of fixed effects, as well as the variance and standard deviation (σ^2^) and number of levels (N) of the random effect. An HR >1 indicates increased hazard of mortality, and <1 indicates decreased hazard of mortality. Reference category of partnership type is no divorce. Effects with a p < 0.05 are in bold.

|  | | A. Females | | | | | B. Males | | | | |
| --- | --- | --- | --- | --- | --- | --- | --- | --- | --- | --- | --- |
| Fixed effects | | β | SE | HR | Z | P | β | SE | HR | Z | P |
| Partnership type | Divorced & lost | **0.847** | **0.245** | **2.332** | **3.46** | **0.001** | -0.089 | 0.336 | 0.915 | -0.26 | 0.790 |
|  | Divorced & kept | 0.612 | 0.388 | 1.844 | 1.58 | 0.110 | -0.003 | 0.352 | 0.997 | -0.01 | 0.990 |
| Maternal age at conception | | 0.073 | 0.066 | 1.076 | 1.11 | 0.270 | 0.055 | 0.061 | 1.057 | 0.91 | 0.36 |
| Random effects | | σ^2^ | N |  |  |  | σ^2^ | N |  |  |  |
| First breeding season ID | | 0.496 | 206 |  |  |  | 0.303 | 224 |  |  |  |
| Mother ID | | 0.020 | 41 |  |  |  | 0.020 | 39 |  |  |  |

Table S10. Output of the Generalized Linear Mixed Model (GLMM) regressing long-term reproductive success (genetic offspring assigned at age >3 years) over whether (A) females (*N*=203) and (B) males (*N*=236) divorced early in life and if so, whether they kept or lost the breeding position in the resident territory. Included are the estimates (β), standard errors (SE), *Z*-value and 95% CI of fixed effects, as well as the variance and standard deviation (σ^2^) of random effects. Effects with p < 0.05 are in bold. Reference of the partnership category is never divorced.

|  | | **A. Females** | | | | **B. Males** | | | |
| --- | --- | --- | --- | --- | --- | --- | --- | --- | --- |
| Fixed effects | | β | SE | z | p | β | SE | z | p |
| Intercept | | 0.759 | 0.061 | 12.527 | <0.001 | 0.712 | 0.083 | 8.524 | <0.001 |
| Longevity | | **0.522** | **0.043** | **12.172** | **<0.001** | **0.663** | **0.045** | **14.832** | **<0.001** |
| Age first reproduction | | -0.004 | 0.050 | -0.081 | 0.936 | -0.084 | 0.051 | -1.662 | 0.097 |
| Partnership type | Divorced & lost position | -0.141 | 0.291 | -0.483 | 0.629 | 0.236 | 0.268 | 0.878 | 0.380 |
|  | Divorced & kept position | -0.088 | 0.221 | -0.396 | 0.692 | -0.508 | 0.344 | -1.477 | 0.140 |
| Random effects | | σ^2^ | N |  |  | σ^2^ | N |  |  |
| First breeding territory ID | | 0.163 | 117 |  |  | 0.351 | 130 |  |  |
| First breeding season ID | | 0.054 | 36 |  |  | 0.292 | 36 |  |  |

Table S11. Output of the Generalized Linear Mixed Model (GLMM) regressing long-term reproductive success (genetic offspring assigned at age >3 years) over whether (A) females (*N*=203) and (B) males (*N*=236) divorced early in life and if so, whether they kept or lost the breeding position in the resident territory excluding longevity as a covariate. Included are the estimates (β), standard errors (SE), *Z*-value and 95% CI of fixed effects, as well as the variance and standard deviation (σ^2^) and number of levels (N) of random effects. Reference of the partnership category is never divorced.

|  | | **A. Females** | | | | **B. Males** | | | |
| --- | --- | --- | --- | --- | --- | --- | --- | --- | --- |
| Fixed effects | | β | SE | z | p | β | SE | z | p |
| Intercept | | 0.889 | 0.082 | 10.813 | <0.001 | 0.764 | 0.123 | 6.200 | <0.001 |
| Age first reproduction | | -0.009 | 0.052 | -0.167 | 0.867 | -0.015 | 0.052 | -0.287 | 0.774 |
| Partnership type | Divorced & lost position | -0.414 | 0.217 | -1.903 | 0.057 | -0.329 | 0.261 | -1.266 | 0.206 |
|  | Divorced & kept position | -0.309 | 0.289 | -1.070 | 0.285 | 0.464 | 0.304 | 1.529 | 0.126 |
| Random effects | | σ^2^ | N |  |  | σ^2^ | N |  |  |
| First breeding territory ID | | 0.163 | 117 |  |  | 0.351 | 130 |  |  |
| First breeding season ID | | 0.054 | 36 |  |  | 0.292 | 36 |  |  |

Table S12. Output of the Generalized Linear Mixed Model (GLMM) regressing number of genetic offspring produced per season from 3 years old until death over whether individuals divorced early in life (first 3 years of life) and if so, whether they kept or lost the resident breeding position for (A) females (*N_obs_*=1522) and (B) males (*N_obs_*=1703). Age of first reproduction (AFR) is the first reproduction after early-life (>3 years). Included are the estimates (β), standard errors (SE), *Z*-value and *p*-value of fixed effects, as well as the variance and standard deviation (σ^2^) and number of levels (N) of random effects. Effects with a p < 0.05 are in bold. Reference category of partnership type is divorced & kept breeding position in resident territory.

|  | | A. Females | | | | B. Males | | | |
| --- | --- | --- | --- | --- | --- | --- | --- | --- | --- |
| Fixed effects | | β | SE | Z | P | Estimate | SE | Z | P |
| (Intercept) | | -1.676 | 0.522 | -3.214 | 0.001 | -0.565 | 0.317 | -1.782 | 0.075 |
| Age | | -0.627 | 0.429 | -1.463 | 0.143 | 0.386 | 0.300 | 1.285 | 0.199 |
| Age^2^ | | 0.066 | 0.233 | 0.281 | 0.779 | **-0.465** | **0.221** | **-2.105** | **0.035** |
| Partnership type | Divorced & lost position | 0.581 | 0.579 | 1.004 | 0.315 | -0.751 | 0.568 | -1.320 | 0.187 |
|  | Never divorced | 0.300 | 0.504 | 0.594 | 0.552 | -0.537 | 0.288 | -1.869 | 0.062 |
| Longevity | | **0.124** | **0.055** | **2.256** | **0.024** | **0.118** | **0.055** | **2.168** | **0.030** |
| Age first reproduction | | -0.044 | 0.048 | -0.925 | 0.355 | **-0.097** | **0.046** | **-2.106** | **0.035** |
| Age * Partnership type: Divorced & lost position | | 0.873 | 0.504 | 1.730 | 0.084 | -0.495 | 0.591 | -0.836 | 0.403 |
| Age * Partnership type: Never divorced | | 0.489 | 0.428 | 1.163 | 0.245 | -0.353 | 0.305 | -1.157 | 0.247 |
| Age^2^ * Partnership type: Divorced & lost position | | -0.229 | 0.326 | -0.703 | 0.482 | -0.258 | 0.765 | -0.337 | 0.736 |
| Age^2^ * Divorced & stayed | | -0.139 | 0.238 | -0.584 | 0.560 | 0.378 | 0.224 | 1.684 | 0.092 |
|  | |  | σ^2^ | N |  |  | σ^2^ | N |  |
| Random effects | | Female ID | <0.0001 | 235 |  | Male ID | 0.276 | 274 |  |
|  | | Territory ID | <0.0001 | 146 |  | Territory ID | <0.0001 | 142 |  |
|  | | Field season ID | 0.841 | 39 |  | Field season ID | 0.835 | 39 |  |
